# Counterfactual quantum-information transfer without transmitting any physical particles

**DOI:** 10.1038/srep08416

**Published:** 2015-02-12

**Authors:** Qi Guo, Liu-Yong Cheng, Li Chen, Hong-Fu Wang, Shou Zhang

**Affiliations:** 1 Department of Physics, Harbin Institute of Technology, Harbin 150001, China; 2 Department of Physics, College of Science, Yanbian University, Yanji, Jilin 133002, China; 3 Department of Applied Physics, Changchun University, Changchun 130022, China

## Abstract

We demonstrate quantum information can be transferred between two distant participants without any physical particles traveling between them. The key procedure of the counterfactual scheme is to entangle two nonlocal qubits with each other without interaction, so the scheme can also be used to generate nonlocal entanglement counterfactually. We here illustrate the scheme by using flying photon qubits and Rydberg atom qubits assisted by a mesoscopic atomic ensemble. Unlike the typical teleportation, the present scheme can transport an unknown qubit in a nondeterministic manner without prior entanglement sharing or classical communication between the two distant participants.

Quantum mechanics predicts many novel counterintuitive effects, such as quantum entanglement, nonlocality, complementarity, and so on. Combined with classical information science, quantum mechanics promotes an interdisciplinary field in recent decades, i.e. quantum information science^1^, which can achieve lots of information processing tasks that appear unimaginable in the classical domain. In quantum information, the minimal unit is qubit, which is usually encoded in the quantum state of a physical entity. Hence the transfer of quantum state carrying quantum information, i.e. quantum information transfer, is the foundation of quantum communication. In 1993, Bennett *et al.* proposed that an unknown quantum state can be teleported to a distant receiver with the help of prior entanglement sharing and classical communication^2^. That scheme, called quantum teleportation, has opened the door for the intense study of quantum communication^3^,^4^,^5^. Another strategy for transferring an unknown quantum state to a distant location can be achieved by using a flying qubit to interact with two spatially separated stationary qubits^6^,^7^,^8^. Note that these kinds of quantum information transfer scheme require the particles carrying information (classical bits or qubits) to travel between the separated participants.

On the other hand, counterfactual quantum information processing has been attracting more and more scientists' attention in recent years. The counterfactuality means relevant quantum information tasks can be achieved without physical particles travelling between two parties. Using a Mach-Zehnder type interferometer, in 1993 Elitzur and Vaidman proposed the information about the existence of an object in a given region of space can be yielded without interacting with it, because an obstructing object in one of the arms of the Mach-Zehnder interferometer could destroy the interference even if no photon was absorbed by the object^9^, which has been called “interaction-free measurements” and improved by Kwiat *et al.*^10^. Subsequently, Kwiat *et al.* proposed that macroscopic entangled states of light can be produced by use of the interaction-free measurement of a quantum object in a quantum superposition state^11^. Using a novel “chained” version of the quantum Zeno effect^12^, Hosten *et al.* proposed quantum computation task can be achieved counterfactually even if the computer is not run^13^. What's more, since Noh proposed the first counterfactual quantum key distribution (CQKD) in 2009^14^, counterfactual quantum cryptography has been studied extensively both theoretically^15^,^16^ and experimentally^17^,^18^. These works showed that secret information could be distributed in a secure way between two remote parties even though no particle transmitted through the quantum channel. In 2013, Salih *et al.* presented that classical bits encoded by the presence and absence of a photon's obstructing object can be transferred counterfactually from the sender to the receiver without any particles traveling between them^19^, which challenged the longstanding assumption that information transfer requires physical particle to be transmitted between two participants. And this work has also attracted much attention^20^,^21^,^22^,^23^,^24^. Recently, we proposed a protocol for counterfactual entanglement distribution by constructing a tripartite nested Mach-Zehnder type interferometer^20^, and demonstrated that the counterfactual distributed controlled-phase gate for quantum dot (QD) spin qubits in double-sided optical microcavities can be implemented^21^.

Inspired by the counterfactual classical information transmission in Ref. 19, in this paper, we examine whether quantum information can be transferred counterfactually without transmitting any physical particles in the practical experiment. Although Salih proposed that an unknown qubit can be transported counterfactually in a deterministic manner^22^, the realistic method to achieve a quantum obstructing object in the superposition state of presence and absence was not detailed. Moreover, the protocol in Ref. 22 required two counterfactual CNOT gates based on “dual” chained quantum Zeno effect and several single-qubit gates, which also increase the circuit complexity and the experimental difficulty. Here we study the counterfactual quantum-information transfer in a more realistic way by employing the dipole blockade between a Rydberg atom and a mesoscopic atomic ensemble, and the results show that the quantum information can be counterfactually transferred to a distant place without transmitting any physical particles with 50% probability, which extremely differs from the typical teleportation protocol. In the scheme, the combined system of the single atom and the mesoscopic atomic ensemble acts as a quantum obstructing object in an unknown quantum superposition state of presence and absence, in which the absorption or passing of the photon depends on the quantum state of the single atom. In principle, as long as the quantum control device can be implemented, our scheme is universal for many physical systems of quantum information processing such as trapped ion systems, superconducting quantum systems, and so on. Though any atom with a transition resonant with the photon's frequency can be used to act as the quantum obstructing object, the scheme cannot be achieved with high probability due to the weak coupling between a single atom and a single photon. However, the atomic ensemble can enhance the coupling strength with the single photon and provide an ideal candidate for the quantum obstructing object. We first introduce the quantum control device using ensemble atoms, then demonstrate an unknown quantum state can be transferred between two distant participants without any physical particles traveling between them.

## Results

### Quantum control device based on Rydberg dipole blockade

Now, we discuss how to control the blocking or passing of photons by a quantum state, which is equivalent to placing the obstructing object of the previous counterfactual schemes in a quantum superposition state of presence and absence. We implement the quantum control device by a Rydberg atomic ensemble. It's well known that atoms excited to high-lying Rydberg states interact with each other via strong and long-range dipole-dipole interaction^25^,^26^, which can block transitions of more than one Rydberg excitation in mesoscopic atomic ensembles, i.e. Rydberg dipole blockade. The mesoscopic ensemble with Rydberg atoms in a blockade radius can be consider as a superatom that all the atoms share a single Rydberg excitation^27^,^28^,^29^,^30^,^31^. The atoms in an ensemble are indistinguishable and have the same emission and absorption properties, hence the interaction between a single photon and an atomic ensemble is a collective process that all the atoms contribute. Therefore, for an ensemble with a mesoscopic number of atoms *K*, the coupling strength between the single photon and the ensemble is 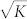 times stronger than that of the photon with a single atom^32^.

**Figure 1:**
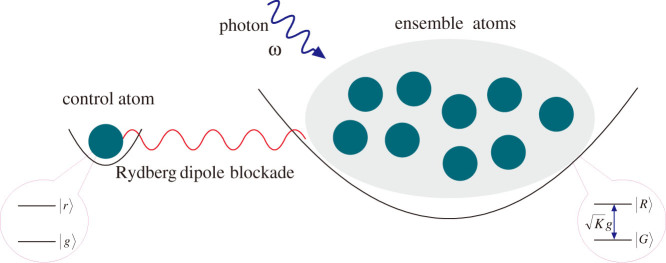
Quantum control device for the passing or blocking of the incident single photon with the frequency *ω*. A control atom and a mesoscopic Rydberg atomic ensemble are stored in two separate trapping potentials, and the ensemble forms a superatom with the collective ground state |*G*〉 and the Rydberg state |*R*〉. The single atom controls the transmission properties of the ensemble by Rydberg dipole interaction. The photon will be absorbed by the ensemble for the control atomic state |*g*〉, and will pass through the ensemble for the control atomic state |*r*〉.

The quantum control device includes a control atom and a mesoscopic Rydberg atomic ensemble stored in two separate trapping potentials respectively as shown in Fig. 1, which has been use to realize Rydberg gate in Refs. 33, 34. All the atoms in the system are identical, and the distance between the two trapping potentials is less than the blockade radius. We encode the qubit in the atomic stable ground state |*g*〉 and the long-lived Rydberg state |*r*〉. The ensemble can be regarded as a superatom with the size of several micrometers and with the collective ground state |*G*〉 and the Rydberg state |*R*〉. We adopt the notation 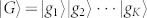 with all the *K* Rydberg atoms in the ground state, and 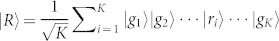 with one and only one atom excited to the Rydberg state. Suppose the transition between the states |*g*〉 and |*r*〉 of the atom is resonant with the photon's frequency *ω*. Initially, the control atom is in the superposition state of |*g*〉 and |*r*〉, and the ensemble is in the collective ground state |*G*〉. If the control atom is in the state |*g*〉, the photon will be absorbed by the ensemble with high probability. However, if the control atom is in the state |*r*〉, it will interact with the ensemble via long-range dipole-dipole forces and lift the energy level of the ensemble's Rydberg state to enable off-resonant transition from |*G*〉 to |*R*〉, which will prevent the ensemble absorbing a photon, and the photon can pass through the ensemble. Therefore, the system in Fig. 1 acts as a quantum version of the obstructing object for a single photon, that is, the blocking or passing of the photon depends on the quantum state of the control atom, which is equivalent to that the obstructing object is in the superposition of presence and absence.

### Interaction-free nonlocal entanglement generation

**Figure 2:**
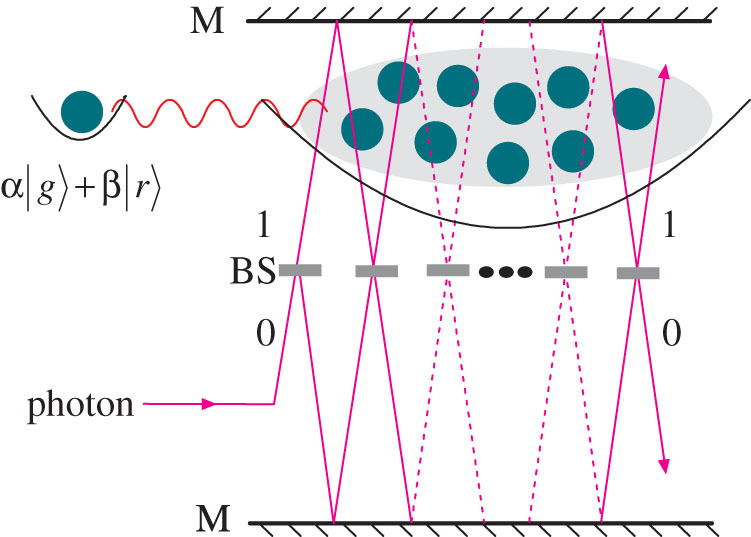
Schematic of interaction-free nonlocal entangled state generation. *N* unbalanced beam splitters BS form a tandem Mach-Zehnder-type interferometer with the two optical pathes 0 and 1. The ensemble is inserted in the path 1, and the single photon enters the interferometer from the path 0. M is normal mirror.

Before discussing the counterfactual quantum information transfer, it is necessary to introduce a method to generate nonlocal photonatom entangled state by repeatedly using a Mach-Zehnder-type interferometer with an atomic ensemble inserted in one of the arms, which is used as the inner interferometer in the following quantum state transfer scheme. The setup is shown in Fig. 2, where M is normal mirror, and BS indicates unbalanced beam splitter with the transmissivity sin^2^ *θ* and the reflectivity cos^2^ *θ*. The *N* BSs form a tandem Mach-Zehnder-type interferometer, and the ensemble is inserted in one of the arms of every interferometer. We encode the photonic qubit in the path degree of freedom, i.e. the lower path is |0〉 and the upper path is |1〉. The control atom is initially prepared in an arbitrary state *α*|*g*〉 + *β*|*r*〉, and the ensemble is in the collective ground state |*G*〉. The photon enters in the interferometer from path 0. The action of the BS can be given by the transformations |0〉 → cos *θ*|0〉 + sin *θ*|1〉 and |1〉 → cos *θ*|1〉 − sin *θ*|0〉. After the first BS, the joint state of the photon and the control atom becomes
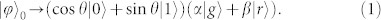
Then the component of the photon in the upper mode will enter the ensemble. For the control atom state |*g*〉, the photon will be absorbed by the ensemble with the transition |*G*〉 → |*R*〉, while it will pass through the ensemble for the state |*r*〉. Therefore, when the photon arrives the second BS, which means the photon component in path 1 was not absorbed, and the quantum state is given by
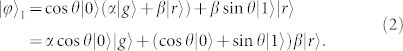
Note that the above state is not normalized, because the component |1〉|*g*〉 is ignored here due to the absorption of the ensemble. In the same way, after *N* cycles, i.e. the photon passes through the *N*th BS and has not been absorbed, the system state becomes
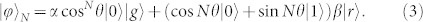
Let *θ* = *π*/2*N*, the final state is
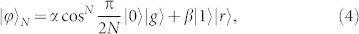
which is a non-maximal hybrid entangled state between the photon and the control atom. The probability of obtaining the entangled state is |*α*|^2^ cos^2*N*^(*π*/2*N*) + |*β*|^2^. Obviously, for the large cycles *N*, the probability will be close to unit and the state will be normalized, |*φ*〉*_N_* ~ *α*|0〉|*g*〉 + *β*|1〉|*r*〉, and for 
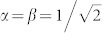
, the maximal entangled state can be obtained. The present protocol may not be the optimal scheme for the entangled state preparation, however, it is worth noting that this is an interaction-free scheme. During the process of the entangled-state generation, although the photon touched the atomic ensemble, it doesn't interact with the ensemble or the control atom at all. From the discussions above, once the photon interacts with the ensemble, it will be absorbed. Therefore, it can be considered as a quantum version of the interaction-free measurement in Ref. 10. From the obtained entangled state, it can be seen when the control atom is initially in |*g*〉, the photon will appear at the lower output port of the tandem interferometer; when the initial state of the control atom is |*r*〉, the photon will exit from the upper output port, which is crucial for the following counterfactual quantum state transfer without any particles traveling in the transmission channel.

### Counterfactual unknown quantum state transfer

**Figure 3:**
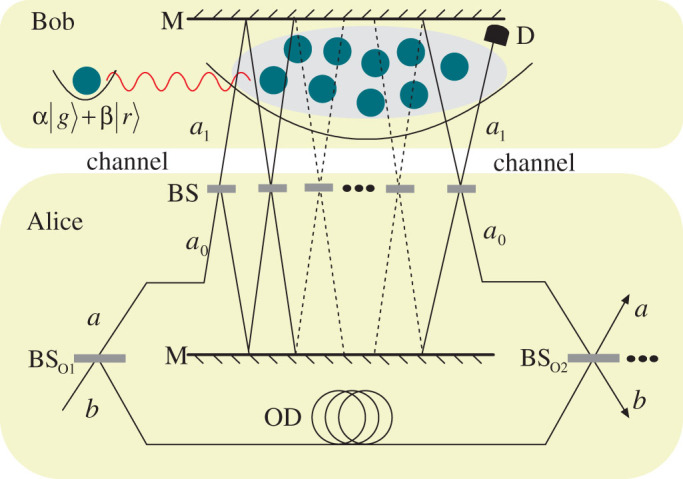
The nested Mach-Zehnder-type interferometer shared by two distant participants, sender Bob and receiver Alice. The interferometer in Fig. 2 as a inner interferometer is nested in one arm of a outer Mach-Zehnder-type interferometer. Connecting *M* such outer interferometers in series, we can implement counterfactual nonlocal entangled state generation and quantum state transfer. The two optical pathes of the outer interferometer are labeled as *a* and *b*, and the two optical pathes of the inner interferometer are *a*_0_ and *a*_1_. OD: optical delay line used to match the optical path lengthes of the different paths of the interferometer. D: conventional photon detector used to absorb the photon exits from the output port of *a*_1_.

**Figure 4:**
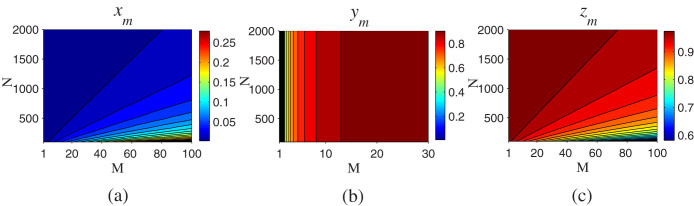
The parameters *x_m_*, *y_m_*, and *z_m_* in Eq. (10) versus the different values of *N* and *M*. (a) *x_m_* is close to zero for large *N* and appropriate *M*. (b) *y_m_* approaches 1 with the increase of *M* and doesn't change with *N*. (c) *z_m_* is close to 1 for appropriate values of *N* and *M*.

Now we show how to counterfactually generate a nonlocal entangled state and transfer an unknown quantum state from Bob to Alice without any physical particles travelling between them. The scheme is accomplished in the ideal limit, by connecting *M* nested Mach-Zehnder-type interferometer in series. Figure 3 shows one nested Mach-Zehnder-type interferometer. The interferometer in Fig. 2, as an inner interferometer, is inserted in one of the arms of an outer Mach-Zehnder-type interferometer. The two optical paths *a* and *b* form the outer interferometer, which means the photon must undergo *N* inner cycles in every outer cycle. BS*_O_* is the outer unbalanced beam splitter with the transmissivity sin^2^ *ϑ* and the reflectivity cos^2^ *ϑ*. Optical delay (OD) is used to match the optical path lengthes of the different paths of the interferometer. Choosing suitable cycle numbers *N* and *M* corresponding to the inner interferometer and outer interferometer respectively, an unknown quantum state transfer can be counterfactually achieved. Suppose the sender Bob wants to transfer an arbitrary quantum state *α*|*g*〉 + *β*|*r*〉 of the control atom to the receiver Alice. Alice sends a photon into the interferometer from the input port *b*. The photon passes through BS*_O_*_1_ that performs the transformations |*a*〉 → cos *ϑ*|*a*〉 + sin *ϑ*|*b*〉 and |*b*〉 → cos *ϑ*|*b*〉 − sin *ϑ*|*a*〉 (*ϑ* = *π*/2*M*), and the joint state of the photon and the atom becomes
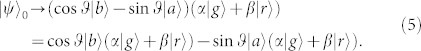
Then the path *b* stays at Alice's site, hence the first term of Eq. (5) is invariant in the outer interferometer. The photon component in path *a* will enter the inner interferometer with the lower path *a*_0_ and upper path *a*_1_ as shown in Fig. 3. Therefore, the evolution of the second term of Eq. (5) in the inner interferometer is the same as the above subsection. After the *N* cycles in the inner interferometer, the system state is given by
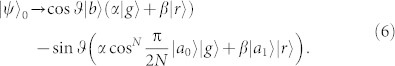
When the photon exits from the inner interferometer, the |*a*_1_〉 component will be absorbed by the detector D. So before the photon reaches the second beam splitter BS*_O_*_2_, the first outer cycle is finished and the state can be written as
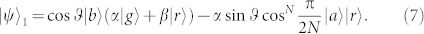
Both Eq. (6) and Eq. (7) are not normalized, because the components absorbed by the ensemble and D can't reach the BS*_O_*_2_ and have been ignored. Through calculation we know when the photon finishes the *m*th (2 ≤ *m* ≤ *M*) outer cycle, i.e. the photon passes through the *m*th outer beam splitter BS*_OM_* and the *m*th inner interferometer, the system state can be written as
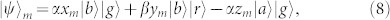
where the parameters *x_m_*, *y_m_*, and *z_m_* satisfy the recursion relations
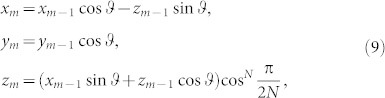
with *x*_1_ = *y*_1_ = cos *ϑ* and *z*_1_ = sin *ϑ* cos*^N^* (*π*/2*N*). We plot the variation trend of the parameters *x_m_*, *y_m_*, and *z_m_* with the values of *N* and *M*, as shown in Fig. 4. It's obvious that *x_m_* → 0, *y_m_* → 1, and *z_m_* → 1 for large values of *N* and *M*, for example, (*x* = 0.0059, *y* = 0.9994, and *z* = 0.9905) for (*M* = 30 and *N* = 2000), which means that after the *M*th outer cycle, the nonlocal hybrid entangled state can be obtained with the probability close to 1. That is
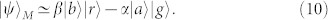
So far, the nonlocal entangled state generation is achieved. Obviously, during the whole process, the probability that the photon travels the channel is nearly suppressed to 0 by repeatedly using the nested Mach-Zehnder-type interferometer. In other words, as long as the photon passes through the channel, if the control atom is in the state |*g*〉, it will be absorbed by the ensemble; while if the atom is in the state |*r*〉, the photon will be absorbed by the detector D that can be seen from Eq. (6). That is to say, as long as the photon appears at the end output port, it has not passed through the transmission channel between Alice and Bob. So this is a counterfactual scheme with no photon passing through the transmission channel.

It's straightway to achieve the quantum state transfer, once the entangled state in Eq. (10) is obtained. Bob performs a Hadamard transformation 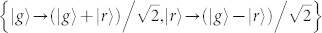 on the control atom state, which can be achieved with a controlled Rabi oscillation with a 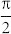 pulse, then the state is given by
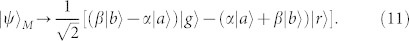
Then Bob detects the atom state in the basis {|*g*〉, |*r*〉}. For the detection result |*r*〉, the teleported state of the atom is perfectly transferred to the path qubit of the photon. If the detection result isn't informed Alice by classical communication, Alice can obtain the teleported state with the probability of 50%, however, if the detection result is sent to Alice, she will perfectly obtain the transferred state with the help of a single-qubit phase flip gate.

It has been shown that an unknown quantum state (or qubit) can be transferred probabilistically without exchanging particle between the two participants. Compared with the typical quantum teleportation, the present scheme doesn't require prior entanglement sharing or even classical communication. Moreover, when the quantum state is transferred to the photon, the initial state of the control atom is destroyed by Bob's detection, which makes the scheme avoid to violate the quantum no-cloning theorem. On the other hand, during the state transfer process, although the photon does not travel to Bob's site, the optical path length it travels is near 2*MN* times that of the distance between Alice and Bob, so the scheme here cannot realize the superluminal communication. Therefore, the present scheme achieves the quantum counterfactuality without contradicting any existing physical law.

## Discussion

Now we analyze and discuss the performance of the quantum information transfer. Obviously, this scheme can be accomplished under the ideal conditions. However, considering the practical experimental implementation of the present scheme, the performance must be affected by the imperfections of the system. One of the basic elements in the present scheme is the composite system of a single control atom and a mesoscopic Rydberg atomic ensemble stored in two separate trapping potentials. There are several advantages using such system. For example, the photon-ensemble coupling is 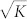 times stronger than that of photon-atom coupling, and the scheme does not require individual addressing of the ensemble atoms. The atoms used in the scheme can be implemented with rubidium or cesium, and the mesoscopic ensemble can be achieved in a cold Rydberg gas with alkali-metal vapor. The existing works shows that the separate trapping potentials can be achieved by many ways, such as two diploe traps^28^,^29^, large-spacing optical lattices^35^, and magnetic trap arrays^36^. The effective manipulation techniques of the atom system has been reported in the previous works^25^,^26^,^27^,^28^,^29^,^30^,^31^,^32^. Especially, the system with a control single atom and a ensemble used in the present scheme has been described in detail in Refs. 33, 34. Therefore, here we mainly focus on the influence factors from other experimental imperfections.

**Figure 5:**
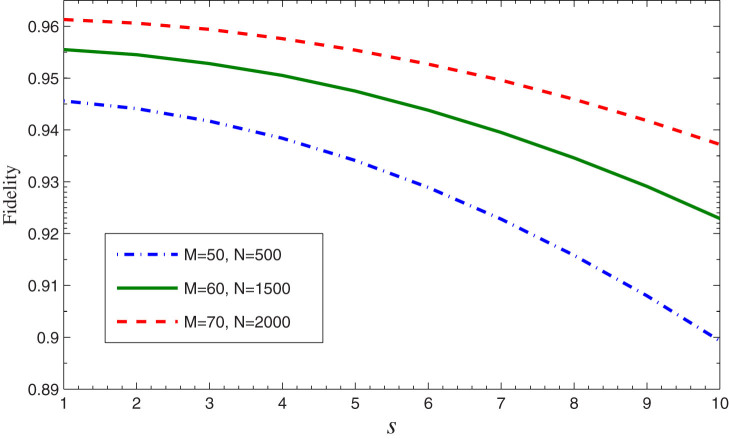
The average fidelity of the counterfactual quantum state transfer versus the error coefficient *s* of the unbalanced beam splitter.

First, the scheme requires high-precision unbalanced beam splitters BS*_s_* (BS*_Os_*) with the transmissivity sin^2^ *θ*(*ϑ*) and the reflectivity cos^2^ *θ*(*ϑ*) (*ϑ* = *π*/(2*M*), *θ* = *π*/(2*N*)), which however are bound to be introduced a slight error in the practical situations. As defined in Ref. 19, we suppose the error coefficients of the inner BSs and the outer BSs are *s*_1_ and *s*_2_ respectively, which indicates the transmissivity and the reflectivity of the each outer (inner) BS has a slight error Δ*ϑ* = *s*_1_*ϑ*/*M* (Δ*θ* = *s*_2_*θ*/*N*). Therefore, we can derive the real final state 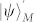 after the *M*th outer cycle by replacing *ϑ* (*θ*) in the recursion relations of Eq. (9) with *ϑ* + Δ*ϑ* (*θ* + Δ*θ*). In order to estimate the influence of the SPRs error in detail, we analyze the average fidelity of the system state after *M* outer cycles. Without loss of generality, let the normalization coefficients *α* and *β* in Eq. (12) equal cos *ξ* and sin *ξ*, respectively. And the average fidelity of the final state can be written as 
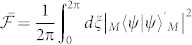
. Assume the error coefficients *s*_1_ = *s*_2_ = *s*, we numerically estimate the average fidelity and plot its change with *s* for different values of *M* and *N* in Fig. 5, which indicates that the fidelity is higher for lesser error factor *s*.

**Figure 6:**
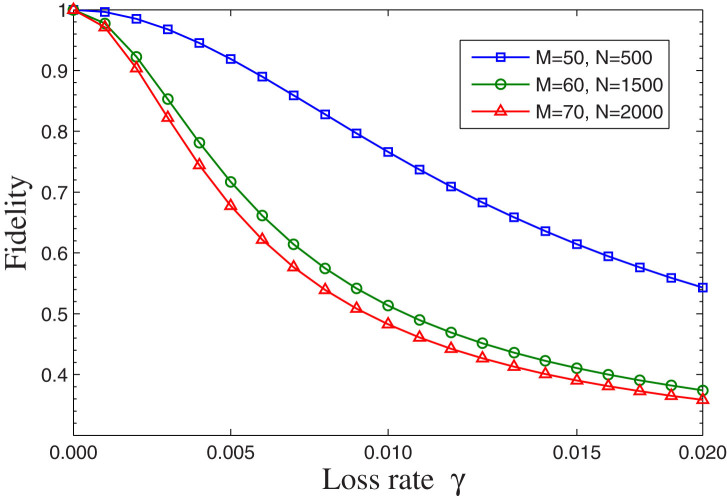
The average fidelity of the counterfactual quantum state transfer versus the photon loss rate *γ* in the transmission channel for different values of *M* and *N*.

Because the single photon may be lost in the transmission channel, hence the photon's loss will affect the efficiency of the quantum information transmission. Especially, the scheme requires large cycle values *M* and *N*, which means the photon's loss is a non-negligible influence factor. We define the photon's loss rate *γ* as the probability that the photon is absorbed by other objects in the transmission channel of every cycle rather than the ensemble or the detector. To quantitatively analyze the effect of the loss, we need to re-derive the quantum-information transfer process with the loss rate *γ*. Obviously, the loss dose not affect the component |1〉|*g*〉, however, the coefficient of the component |1〉|*r*〉 in Eq. (2) will become (1 − *γ*)*β* sin*θ*, which indicates that even though the photon will not be absorbed by the ensemble for the atom state |*r*〉, it may be lost in the channel with the probability *γ*. We can evaluate the effect of the photon loss on the average fidelity of quantum information transfer to qualify the influence of the loss. The numerical simulation of the average fidelity for different values of *γ*, *M*, and *N* is shown in Fig. 6, which shows that the present scheme is sensitive to loss and has higher fidelity when the loss probability is suppressed under about 0.5%. For example, it can be calculated when *γ* = 0.2%, *M* = 60, and *N* = 1500, the fidelity 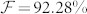. It also shows that the effect of the photon loss is more obvious for larger values of *M* and *N*.

In summary, we have proposed a counterfactual scheme for transferring an unknown quantum state without transmitting any physical particles. The scheme indicated that a qubit can be teleported to a distant place without prior entanglement sharing and classical communication between the two distant participants with the probability of 50%, so it essentially differs from the typical teleportation. We also numerically estimated the effect of the imperfections of the experiment system, which indicated our scheme may be feasible under the current technology.

## Methods

### The interaction between a single photon and a mesoscopic atomic ensemble

Due to the dipole blockade, the ensemble with *K* Rydberg atoms in the present scheme can be considered as a superatom with two collective energy levels, i.e. the ground state 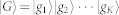 and the Rydberg state 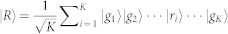. Therefore, the interaction of the single photon and the ensemble can be described by the Hamitonian
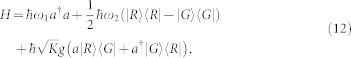
where *ω*_1_ is the single photon frequency, and 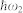 represents the energy difference between the ground state |*g*〉 and the Rydberg state |*r*〉 of the single atom. *g* is the coupling strength of a single photon and a single atom, hence the photon-ensemble coupling strength is 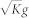. For the resonant case, i.e. *ω*_1_ = *ω*_2_ = *ω*, the Hamitonian in the interaction picture is given by 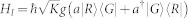. The ensemble is initially in the collective ground state |*G*〉, and the Hamitonian will function for the control atom in the state |*g*〉.

## Author Contributions

Q.G. designed the scheme and wrote the manuscript under the guidance of H.F. and S.Z.. Q.G., L.Y. and L.C. carried out the theoretical analysis. All authors contributed to the interpretation of the work and the writing of the manuscript. All authors reviewed the manuscript.
